# Diagnosis of a patient with severe sensorineural hearing loss as the initial symptom caused by novel compound heterozygous variant in SLA19A2 gene

**DOI:** 10.1016/j.bjorl.2025.101581

**Published:** 2025-04-11

**Authors:** Yanan Shi, Junyang Li, Xiaoqin Chen, Niu Li, Sijie Yang, Youjin Li, Min Zhou

**Affiliations:** a Shanghai Jiao Tong University, School of Medicine, Shanghai Children’s Medical Center, Hainan Branch, Department of Otolaryngology, Sanya, China; b Shanghai Jiao Tong University, School of Medicine, Shanghai Children’s Medical Center, Department of Otolaryngology, Shanghai, China; c Fujian Medical University, College of Clinical Medicine for Obstetrics & Gynecology and Pediatrics, Fuzhou, China; d Shanghai Jiao Tong University, School of Medicine, The International Peace Maternity and Child Health Hospital, Shanghai, China; e Shanghai Jiao Tong University, School of Medicine, Shanghai Children’s Medical Center, Key Laboratory of Pediatric Hematology and Oncology of China Ministry of Health, Department of Hematology and Oncology, Shanghai, China; f Shanghai Jiao Tong University, School of Medicine, Shanghai Children’s Medical Center, Hainan Branch, Department of Hematology and Oncology, Sanya, China

**Keywords:** Thiamine-responsive megaloblastic anemia syndrome, Sensorineural hearing loss, SLC19A2 gene, Novel compound heterozygous variant, Functional study

## Abstract

**Objective:**

Thiamine-Responsive Megaloblastic Anemia (TRMA) syndrome, caused by biallelic variants in the SLC19A2 gene, typically presents with a triad of megaloblastic anemia, diabetes mellitus, and sensorineural hearing loss. This study aims to determine the genetic etiology and clinical phenotype of a patient who presented with severe sensorineural hearing loss as the initial symptom, and to expand our understanding of the SLC19A2 variant spectrum.

**Methods:**

Proband-only whole-exome sequencing was performed to screen the candidate variants, which were subsequently validated by Sanger sequencing within the family. cDNA sequencing based on RT-PCR and TA cloning analysis was used to determine the effect of splicing variants on mRNA processing of SLC19A2 gene. Detailed clinical features were evaluated by a diagnostic hearing test, laboratory and imaging examination.

**Results:**

A 2-year-5-month-old Chinese girl was diagnosed with diabetes mellitus and severe sensorineural hearing loss, without abnormal hemoglobin. DNA sequencing revealed a novel compound heterozygous variant of c.808-1G > A and c.1228C > T (p.Gln410*) in the SLC19A2 gene. Both variants were previously unreported. The c.808-1G > A splicing variant is located in intron 2 of SLC19A2, and is predicted to cause exon 3 skipping. The cDNA experiment confirmed this biological event, further indicating that the splicing variant can cause amino acid frameshift alteration (p.Glu270Valfs*10) in SLC19A2.

**Conclusion:**

We report a patient with TRMA syndrome (without anemia) caused by a novel compound heterozygous variant in SLA19A2 gene. This study suggests that the possibility of TRMA syndrome should be considered when encountering patients with early-onset severe sensorineural hearing loss in clinical practice.

**Level of evidence:**

Level 4.

## Highlights

• Identified novel compound heterozygous variants in SLC19A2 causing TRMA syndrome.
• Severe sensorineural hearing loss was the initial symptom in a TRMA syndrome case.
• cDNA analysis confirmed exon 3 skipping and frameshift mutation in SLC19A2.
• TRMA syndrome should be considered in patients with hearing loss and glucose issues.
• Findings expand the pathogenic variant spectrum of SLC19A2 in TRMA syndrome.

## Introduction

Thiamine-Responsive Megaloblastic Anemia syndrome (TRMA, OMIM# 249270) is a rare recessive condition caused by variants in the Solute Carrier family 19 member 2 (SLC19A2) gene.^1^ The SLC19A2 gene is located on chromosome 1q23.3 and encodes a High-affinity Thiamine Transporter-1 (THTR-1), which is the main thiamine transporter in many tissues and is critical to cell survival and function. Disruption of this transporter can lead to a decrease in intracellular thiamine concentrations, resulting in apoptosis and dysfunction of cochlear cells, islet cells, and hematopoietic stem cells. Accordingly, TRMA patients may present with megaloblastic anemia, progressive sensorineural hearing loss and non-autoimmune diabetes.^2^, ^3^, ^4^ In addition, some affected individuals (less than 30%) may also exhibit other congenital abnormalities such as ophthalmologic, cardiovascular, neurologic abnormalities, and thrombocytosis.^5^ Notably, the majority of reported TRMA patients have ancestral origins in the Middle East, South Asia, and the Northern Mediterranean region, with more than 60% being homozygous for SLC19A2 due to consanguinity among their parents.^6^

In this study, we reported a case of a 2-year-old and 5-month-old Chinese girl with severe sensorineural hearing loss as the prominent and initial symptom. Genetic sequencing revealed a novel compound heterozygous variant in SLC19A2 that can explain the condition of the patient. We yet conducted in vivo functional studies to further analyze the pathogenicity of the novel splicing variant.

## Methods

### Patient

A 2-year-5-month girl, who was found with hearing loss for over 3-months, was referred to the Otolaryngology Department of our hospital. A comprehensive evaluation was conducted on the child, including inquiring about the current medical history, family history, physical examination, hearing test, laboratory examination, and imaging examination. Audiological evaluations, including tympanometry, Otoacoustic Emissions (OAE), Auditory Steady-State Response (ASSR), and Auditory Brainstem Response (ABR) were performed according to standard protocols.

### Whole-exome sequencing

Proband-only Whole-Exome Sequencing (WES) was performed in the family and the experimental procedure was described previously.^7^ Briefly, genomic DNA was extracted from the proband’s peripheral blood and sheared to create fragments of 150 to 200bp. Sequencing library was prepared using the SureSelect XT Human All Exon V6 reagent kit (Agilent Technologies, Santa Clara, CA, USA), and high-throughput sequencing was performed with the Illumina NovaSeq 6000 System (Illumina, San Diego, CA, USA). Original sequencing data were assessed using FastQC (version 0.11.2) for quality assessment and were then aligned to a reference human genome (Human 37.3; SNP135) by Burrows-Wheeler Alignment tool (BWA, v0.2.10). All the single nucleotide variants (SNVs) and indels identified by GATK were uploaded to TGex™ (LifeMap Sciences, Inc. Walnut, CA, USA) software for biological analysis and interpretation.

### Sanger sequencing verification of the SLC19A2 gene

The primers to amplify the SLC19A2 gene (GenBank Accession nº NM_006996.3) were designed using Primer 3 software (http://primer3.ut.ee/). Primers designed for exon 3 were forward: 5′-GAAGCCTGCGGTCTCTTTTC-3′ and reverse: 5′-TGGGAGGGGTGAATAAATCTGA-3′. Primers designed for exon 5 were forward: 5′-TCCCATGTTTGTATGTTCCTACT-3′ and reverse: 5′-TGAAGGAAGGAGGGTATGCT-3′. The targeted exons and exon-intron boundaries were amplified via polymerase chain reaction. Sanger sequencing was performed by ABI 3500 sequencer (Applied Biosystems, Foster City, CA, USA) to analyze the PCR products.

### Reverse transcription PCR (RT-PCR) and TA-clone sequencing

Total RNA was extracted using TRIzol™ (Thermo Fisher Scientific, Waltham, MA, USA) from the Peripheral Blood Mononuclear Cells (PBMCs) isolated from the proband and her parents using Ficoll-Paque plus (GE, Fairfield, CT), respectively. cDNA was then synthesized using the Hifair® AdvanceFast 1st Strand cDNA Synthesis Kit (Yeasen, Shanghai, China). The following primers were used to amplify a 463-bp fragment of the wild-type SLC19A2: forward (located on exon 2), 5′−CCTATGCCACAGAAGAGCCT-3′; reverse (located on exon 4), 5′-CTCCCCAAGTTGACCAGGAT-3′. GAPDH was used as a control to assess RNA quality of each sample, and the following primers were used to amplify the GAPDH fragment: forward, 5′-GAGATCCTTCCAAAATCAAGT-3′ and reverse, 5′-GTGGAGGAGTGGGTGTCG-3′. Gel electrophoresis was performed to examine the obtained PCR products. The WT and mutant SLC19A2 fragments were then cloned into the pESI-T vector (Yeasen), respectively, and were transformed into *Escherichia coli* DH5α reference strain. Sequencing of the clones was conducted for further identification of the PCR products.

## Results

### Clinical description of the patient

**Fig. 1:**
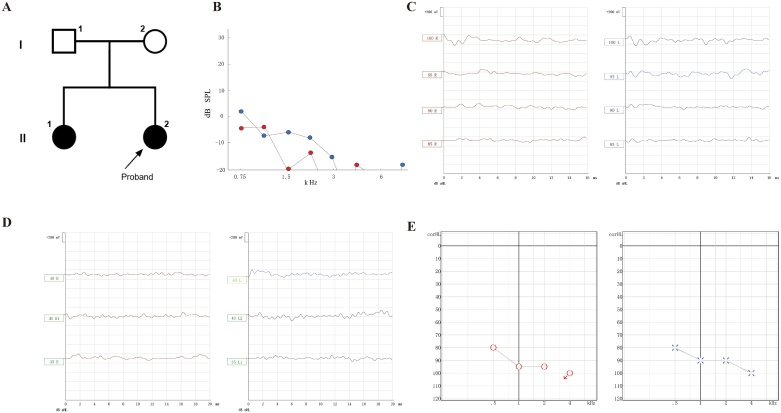
The results of hearing examination of the patient. (A) Pedigree of the family. Males are indicated by squares and females are indicated by circles. Generations are labeled using Roman numerals (I, II). Solid symbols indicate affected individuals. Arrow indicates the proband. (B) Upon conducting the Otoacoustic Emissions (OAE) test, no responses were elicited at any frequency in either ear. (C) Air conduction Auditory Brainstem Response (ABR) showed the threshold in both ears was 95 dB nHL. (D) No response in both ears at 40 dB nHL intensity upon bone conduction ABR test. (E) Auditory Steady-State Response (ASSR) test showed no response in the right ear at sound intensities above 95 dB nHL, whereas the left ear exhibited a response even under high-intensity stimulation. Red and blue indicate thresholds for the right and left ears, respectively.

The proband was the second child born from healthy nonconsanguineous Chinese parents (Fig.1A, II-2). She was vaginally delivered at full term without any special. She did not possess any high-risk factors for hearing loss and had passed the newborn hearing screening. Distortion product otoacoustic emission showed no response in both ears (Fig. 1B). Air conduction ABR test revealed that the thresholds for both the right and left ears were 95 dB nHL (Fig. 1C). Bone conduction ABR showed no response in both ears at 40 dB nHL intensity (Fig. 1D). ASSR test showed that the right ear responds to high-intensity sound stimulation to a certain extent, but there is no response when the sound intensity exceeds 95 dB nHL, while the left ear can still produce ASSR response to high-intensity stimulation (Fig. 1E). The patient was therefore diagnosed with bilateral sensorineural hearing loss according to these diagnostic hearing test results.

When she was 1-year-old, a routine blood test showed that her Mean Corpuscular Volume (MCV) was at the upper end of the reference range (100.4 fL, reference range: 80–100.4 fL), and the hemoglobin level was near the lower limit of the reference range (118 g/L, reference range: 120–160 g/L). Subsequent tests conducted after she turned 2 continued to reveal elevated MCV values (ranging from 94.4–95.2 fL, reference range: 72–86 fL). Both her hemoglobin levels and red blood cell counts were normal (Table 1). Additionally, the patient exhibited irregular glucose metabolism. Both her fasting blood glucose level of 8.27 mmoL/L (reference range: < 5.6 mmoL/L) and 2 -h postprandial blood glucose level of 9.49 mmoL/L (reference range: < 7.8 mmoL/L) were elevated. Additionally, her glycated Hemoglobin (HbA1c) level was 9.60% (reference l range: 4%‒6%), and her urine glucose test result was positive.

**Table 1:** Anemia related laboratory test results.

Test item	Age at examination	Value	Reference range
Red blood cell count	1-year	3.62 × 10^9^/L	3.50‒5.20 × 10^9^/L
	2-year	3.86 × 10^9^/L	4.00–5.50 × 10^9^/L
	2-year‒7-month	3.99 × 10^9^/L	4.00–5.50 × 10^9^/L
	2-year‒9-month	4.56 × 10^9^/L	4.00–5.50 × 10^9^/L
Hemoglobin	1-year	118 g/L	120–160 g/L
	2-year	121 g/L	107‒141 g/L
	2-year‒7-month	118 g/L	112‒149 g/L
	2-year‒9-month	130 g/L	112‒149 g/L
Mean corpuscular volume	1-year	100.4 fL	80.0‒100.0 fL
	2-year	95.2 fL	76.0–88.0 fL
	2-year‒9-month	91.0 fL	76.0–88.0 fL

The remaining laboratory tests revealed no significant abnormalities. Imaging studies, including high-resolution Computed tomography (CT) of the temporal bone and Magnetic Resonance Imaging (MRI) of the inner ear, showed no structural deformities or malformations of the inner ear or auditory nerve.

### Identification of SLC19A2 compound variants

**Fig. 2:**
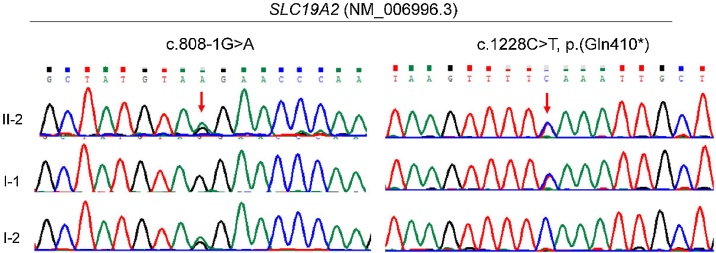
Genetic sequencing results of the family. Sanger sequencing confirmed the patient harbored the compound heterozygous variant of c.808-1G > A and c.1228C > T (p.Gln410*) in the SLC19A2 gene (NM_006996.3), which were inherited from the mother and father, respectively. Red arrows indicate the variant base.

The proband was diagnosed with severe sensorineural hearing loss and was therefore performed with WES to screen for the disease-causing gene. We initially excluded the common variants with Allele Frequencies (AF) greater than 1% in the gnomAD database (http://gnomad.broadinstitute.org/), the harmless missense variants predicted by REVEL^8^ and the variants predicted to have no impact on mRNA splicing by SpliceAI.^9^ Subsequently, clinical symptoms of hearing loss served as filtering indexes to analyze the candidate variants. Eventually, WES revealed that the patient harbored a novel compound heterozygous variant in the SLC19A2 gene, which was validated by Sanger sequencing (Fig. 2). One was a canonical splicing variant (NM_006996.3: c.808-1G > A) in intron 2. The other was a nonsense variant (NM_006996.3: c.1228C > T, p.Gln410*) in exon 5. Both variants were absent in the gnomAD database and have not been reported previously. Sanger sequencing revealed that the splicing variant was inherited from his mother and that the nonsense variant was inherited from his father. According to the clinical interpretation of genetic variants by the ACMG/AMP 2015 guideline,^10^ both variants were classified as pathogenic (PVS1+PM2_supporting + PM3). Additionally, no questionable Copy Number Variations (CNVs) were found Copy Number Variation (CNV) analysis by bioinformatics analysis using the WES data.

### The c.808-1G > A variant results in exon 3 skipping of SLC19A2 at transcription level

**Fig. 3:**
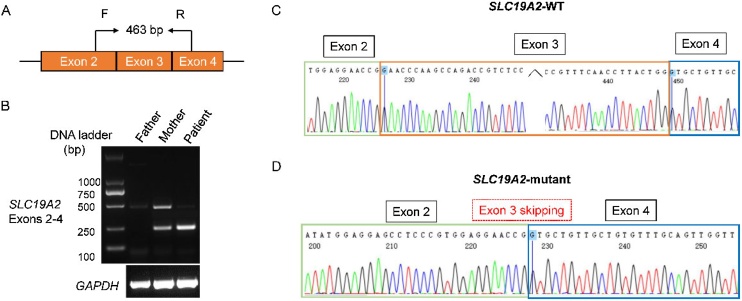
Splicing assays of the c.808-1G > A variant. (A) Schematic representation to show the location of the primers and the expected size of the PCR fragment in Wild-Type (WT) sample. (B) Electrophoresis was used to analyze the RT‑PCR results. A novel smaller band was observed in the patient and her mother’s samples. (C) Direct sequencing confirmed that the expected 463-bp band results from normal splicing of exons 2–4 in the SLC19A2 gene. (D) Clone sequencing revealed that the formation of the novel smaller band due to exon 3 skipping of SLC19A2.

To determine how the c.808-1G > A variant affects mRNA splicing of SLC19A2 gene, the total RNA was extracted from the patient and his parents, respectively, and a pair of primers were designed to amplify the cDNA fragments (Fig. 3A). The expected 463-bp PCR product can be amplified in all three samples, however, there is an additional smaller band in samples of the patient and the mother (c.808-1G > A carrier) (Fig. 3B). TA-clone sequencing showed that the 463-bp PCR product is a cDNA fragment formed by normal splicing of exons 2–4 (Fig. 3C), while the entire exon 3 (223 bp) is missing in the smaller band indicating that the c.808-1G > A variant leads to exon 3 skipping of the SLC19A2 gene (Fig. 3D). Sequence analysis indicates that the deletion of exon 3 will result in an amino acid frameshift alteration (p.Glu270Valfs*10). According to the ACMG guidelines,^10^ the c.808-1G > A variant was classified as pathogenic using the evidence clauses of PVS1, PM2-supporting, and PP4.

### Treatment of hearing loss

In addition to standard thiamine supplementation therapy, the patient also received cochlear (MED-EL, Innsbruck, Austria) implant surgery treatment. Four months post- surgery, the patient completed a period of auditory conditioning training at home and participated in auditory training exercises in a sound field environment. Hearing tests revealed a threshold of 35 dB HL at 1000 Hz in the left ear and 25 dB HL at 1000 Hz in the right ear.

## Discussion

This study reported the genetic etiology and clinical manifestations of a female patient with severe bilateral sensorineural hearing loss and diabetes mellitus. Physical examination showed no abnormalities, and laboratory test results showed a significant increase in red blood cell volume, but no obvious anemia has yet appeared. WES revealed the patient harbored a novel compound heterozygous variant in the SLC19A2 gene. One is the c.808-1G > A splicing variant inherited from the mother, which has been confirmed to cause loss of exon 3 and premature formation of the stop codon. Another is the nonsense variant c.1228C > T (p.Gln410*) inherited from the father, which may lead to nonsense mediated mRNA decay. Based on the clinical manifestations, laboratory tests, and molecular results, the patient was diagnosed with TRMA syndrome. After receiving cochlear implantation treatment, we have observed significant improvement in hearing function.

Thiamine, also known as vitamin B1, is a water-soluble vitamin in the human body that is almost entirely consumed through diet. It is widely present in skeletal muscle, myocardium, liver, kidneys, and brain, and is a key cofactor involved in energy metabolism.^11^ In addition to THTR-1, thiamine can also be transported into cells through passive transport and high affinity Thiamine Transporter-2 (THTR-2, encoded by SLC19A3).^12^ These two transporters are widely expressed in human tissues, and the intracellular thiamine deficiency caused by THTR-1 mutation can be compensated by THTR-2 and passive transport in most tissues.^13^, ^14^ However, THTR-1 is the only known transporter in bone marrow cells, pancreatic beta cells, and some cochlear cells, which is the etiology of the clinical typical triad of TRMA syndrome.^15^ However, it should also be noted that if not identified early and treated promptly (i.e., supplementing with vitamin B1), these patients may develop more severe clinical phenotypes and may involve multiple systems, such as bone marrow failure, neurodevelopmental disorders (microcephaly, cerebral infarction), horizontal nystagmus and hepatomegaly, etc., if not identified and intervened for treatment in a timely manner.^16^, ^17^

Megaloblastic anemia is one of the main characteristics of TRMA syndrome, which usually occurs in infancy and early childhood. Some patients even require multiple blood transfusions for correction. Megaloblastic or circular sideroblastic granulocytes can be observed upon bone marrow biopsy. Diabetes mellitus is another major manifestation of TRMA syndrome, which usually appears at an early stage (the median age of onset is 2 years old). It is mainly manifested as non-type I diabetes with reduced insulin secretion. Sensory sensorineural hearing loss can be the earliest symptom of onset, and hearing loss often appears in childhood and progressively worsens. These patients usually have no structural abnormalities in the inner ear, but thiamine deficiency can cause selective atrophy of inner hair cells, and due to the inability of mature mammalian inner hair cells to regenerate, it will ultimately lead to permanent sensorineural hearing loss.^6^, ^18^ It has been observed that over 90% of patients will eventually exhibit typical triad symptoms, but the onset time of these symptoms may vary. Indeed, some affected individuals have megaloblastic anemia and diabetes at an early age, but no hearing loss.^4^, ^6^, ^19^ For the patient in this study, she had signs of sensorineural hearing loss and diabetes mellitus, but no anemia. In addition, the phenotype of TRMA syndrome is highly overlapping with Wolfram syndrome (caused by variants in CISD2 or WFS1 gene), which is mainly manifested by diabetes, optic atrophy and deafness.^20^, ^21^, ^22^ These results indicate that identifying and accurately diagnosing TRMA syndrome in the early stage may face some challenges, therefore, introducing genetic test approaches can effectively improve diagnostic efficiency.

The main treatment for TRMA syndrome is oral supplementation of vitamin B1 and symptomatic management. Vitamin B1 supplementation can activate other low affinity thiamine transporters, thus delaying or preventing megaloblastic anemia and non-autoimmune diabetes symptoms. However, withdrawal of vitamin B1 may lead to recurrence, so lifelong replacement therapy is often required. However, vitamin B1 supplementation therapy has limited effect on improving hearing loss, possibly because cochlear cells are highly sensitive to thiamine deficiency, and hearing loss may begin to manifest during fetal development. Therefore, patients often need to use hearing aids or cochlear implants to improve their hearing according to the severity of their hearing loss. Some studies have pointed out that thiamine may be effective in preventing deafness,^19^, ^23^, ^24^ nevertheless, such a conclusion still requires more case studies.

## Conclusion

We diagnosed a case of TRMA syndrome presenting with severe sensorineural hearing loss as the initial symptom and identified a novel compound heterozygous variant in the SLC19A2 gene, thereby expanding the known pathogenic variant spectrum of this gene. Our findings suggest that clinicians should consider TRMA syndrome in patients with sensorineural hearing loss, even in the absence of inner ear developmental abnormalities. Special attention should be given to those with concurrent blood glucose abnormalities, anemia, or other systemic symptoms, as early diagnosis and intervention could significantly improve outcomes.

## Ethical approval

The study protocol was approved by the Committee on Human Research of Hainan Hospital of Shanghai Children’s Medical Center Affiliated to Shanghai Jiaotong University School of Medicine (SYFYIRB(K)2024090).

## Funding

The study was supported by a grant from Chinese National Natural Science Foundation (82071015), the 10.13039/501100003399Science and Technology Commission of Shanghai Municipality (21Y11900200 and 22S31905600).

## Declaration of competing interest

The authors declare no conflicts of interest.
